# Camel milk or silymarin could improve the negative effects that experimentally produced by aflatoxin B1 on rat’s male reproductive system

**DOI:** 10.1186/s12917-024-03965-5

**Published:** 2024-03-18

**Authors:** Nahla H. Hassaneen, Shabaan A. Hemeda, Abeer F. El Nahas, Sabreen E. Fadl, Eman M. El-diasty

**Affiliations:** 1 Department of Animal Husbandry and Animal Wealth Development, Faculty of Veterinary Medicine, Matrouh University, Matrouh, Egypt; 2 Department of Animal Husbandry and Animal Wealth Development, Faculty of Veterinary Medicine, Alexandria University, Alexandria, Egypt; 3 Biochemistry Department, Faculty of Veterinary Medicine, Matrouh University, Matrouh, Egypt; 4 Mycology Department, Animal Health Research Institute Dokki, Giza (ARC), Egypt

**Keywords:** Aflatoxin B1, Rat’s reproductive organs, Body weight, Testosterone, Camel milk, Silymarin, Gene expression

## Abstract

**Background:**

Camel milk and silymarin have many different beneficial effects on several animal species. Meanwhile, Aflatoxins are mycotoxins with extraordinary potency that pose major health risks to several animal species. Additionally, it has been documented that aflatoxins harm the reproductive systems of a variety of domestic animals. The present design aimed to investigate the impact of aflatoxin B1 (AFB1) on rat body weight and reproductive organs and the ameliorative effects of camel milk and silymarin through measured serum testosterone, testes pathology, and gene expression of tumor necrosis factor (*TNF-α*), luteinizing hormone receptor (*LHR*), and steroidogenic acute regulatory protein (*StAR*) in the testes. A total of sixty mature male Wister white rats, each weighing an average of 83.67 ± 0.21 g, were used. There were six groups created from the rats. Each division had ten rats. The groups were the control (without any treatment), CM (1 ml of camel milk/kg body weight orally), S (20 mg silymarin/kg b. wt. suspension, orally), A (1.4 mg aflatoxin/kg diet), ACM (aflatoxin plus camel milk), and AS (aflatoxin plus silymarin).

**Results:**

The results indicated the positive effects of camel milk and silymarin on growth, reproductive organs, and gene expression of *TNF-α*, *LHR*, and *StAR* with normal testicular architecture. Also, the negative effect of AFB1 on the rat’s body weight and reproductive organs, as indicated by low body weight and testosterone concentration, was confirmed by the results of histopathology and gene expression. However, these negative effects were ameliorated by the ingestion of camel milk and silymarin.

**Conclusion:**

In conclusion, camel milk and silymarin could mitigate the negative effect of AFB1 on rat body weight and reproductive organs.

## Background

Camel milk is a natural remedy with potent anti-inflammatory properties that have been demonstrated in-vitro and in vivo studies [1]. Camel milk has the highest concentration of bioactive lactoferrin (220 mg/L) when compared to the milk of other species, such as buffalo, cow, goat, sheep, and mare. This substance has unique anti-inflammatory and antioxidant properties [2]. However, research has been conducted on camel milk’s nutritional and therapeutic benefits [3]. Camel milk is a valuable food source that contains bioactive ingredients that may fight off illness and improve well-being [4]. Since ancient times, camel milk has been recognized to have medical properties due to its increased hepatoprotective effect, insulin-like peptides, antibacterial and antiviral activity, as well as anti-cancer, hypoallergenic, antioxidant, antihypertensive, and anti-diabetic characteristics [5]. In a previous study, Govindasamy et al. [6] discovered that giving camel milk to obese diabetic patients may be beneficial. Moreover, Zakaria et al. [7] reported the positive effect of camel milk on male rat reproductive organs.

Silymarin is a polyphenolic flavonoid obtained from the milk thistle. The majority of silymarin’s phytochemical characteristics are due to silybin, which is the primary component of silymarin. Numerous investigations have shown that silymarin has anti-inflammatory, anti-cancer, antifibrotic, and immunomodulatory properties [8]. Moreover, silymarin in rats increases the production of testosterone, luteinizing hormone (LH), follicle stimulating hormone (FSH), gonadotropin-releasing hormone (GnRH), and the number of spermatids and spermatozoa cells [9].

Aflatoxins are poisonous substances made by the *Aspergillus* species mold as a secondary metabolite that mainly contaminates food in tropical regions [10]. The most dangerous and prevalent form of aflatoxins in human populations is aflatoxin B1 (AFB1). Hepatotoxicity and carcinogenesis have been connected to AFB1-exo-8,9-epoxide, the highly reactive metabolite of AFB1 that binds to DNA and creates adducts [11]. AFB1 can cause liver injury, growth inhibition, and oxidative stress [12, 13]. Moreover, AFB1 is highly cancerous and mutagenic to DNA [14]. There is growing concern that exposure to toxic pollutants disrupts male reproduction in both humans and animals and is a major factor in the decline of both the amount and quality of human sperm in recent years [15, 16]. Particularly in males, exposure to AFB1 can change various aspects of reproduction, including sperm production, reproductive function, and testicular and epididymal morphology. The blood hormone levels, which are most notable for a decline in testosterone, represent the most important observation [17]. AFB1 interferes with the hypothalamic-pituitary-testicular axis, which leads to the generation of spermatozoa with defects [18].

The current study investigated the effects of AFB1 exposure on male rats’ body weight, testosterone, testicular pathology, and the expression of certain testicular genes (*TNFα*, tumor necrosis factor α; *LHR*, luteinizing hormone receptor; *StAR*, steroidogenic acute regulatory protein). Along with the ameliorative effects of silymarin and camel milk on the studied parameters.

## Results

The effect of camel milk and silymarin on body weight for rats fed on diets containing 1.4 mg of aflatoxin B1/kg diet for 21 and 28 days is shown in Table (1). At 21 days, the CM, S, A, ACM, and AS groups’ body weight was considerably lower than that of the control group. Meanwhile, the AS group significantly (*P* ≤ 0.05) increased body weight compared with the A group. Regarding the results of body weight at 28 days, compared to the other groups, there was a considerable drop in body weight in the A group. Additionally, the ACM and AS groups’ body weights were much higher than those of the A group. On the other hand, in results from different periods inside the same group, there was a significant (*P* ≤ 0.05) increase in the control group, CM, S, A, ACM, and AS groups at 28 days when compared with results at 21 days.

**Table 1:** Effect of camel milk and silymarin on body weight for rats fed on a diet containing 1.4 mg of aflatoxin B1/kg diet for 21 and 28 days (*n* = 5 for each period/group)

Groups items	Day	Control	CM	S	A	ACM	AS
Initial weight (g)	21	83.2 ± 0.44	84.0 ± 0.47	84.2 ± 0.37	84.1 ± 0.62	83.2 ± 0.60	84.0 ± 0.32
	28	84.0 ± 0.35	83.4 ± 0.51	84.0 ± 0.42	84.1 ± 0.33	84.1 ± 0.24	84.2 ± 0.46
Final weight (g)	21	130.0 ± 1.9^aY^	118.6 ± 0.68^cY^	118.4 ± 0.75^cY^	117.6 ± 1.96^cX^	117.0 ± 1.76^cY^	123.6 ± 2.14^bY^
	28	147.8 ± 0.38^aX^	145.5 ± 0.22^abX^	146.64 ± 1.21^abX^	121.0 ± 1.0^dX^	140.0 ± 1.73^cX^	143.2 ± 1.74^bcX^
Mortality	21	0	0	0	0	0	0
	28	0	0	0	0	0	0

Values are means ± SE. Mean values with different subscript letters (a–d) at the same row significantly differ at (*P* ≤ 0.05). Mean values with different subscript letters (X-Y) at the same column significantly differ at (*P* ≤ 0.05). CM: Camel milk; S: silymarin, A: Aflatoxin, ACM: Aflatoxin + camel milk, AS: Aflatoxin + silymarin

The effect of camel milk and silymarin on serum testosterone for rats fed on diets containing 1.4 mg of aflatoxin B1/kg diet for 21 and 28 days is shown in Table (2). The serum testosterone concentration significantly (*P* ≤ 0.05) decreased in the A group when compared with the control group at 21 days. Meanwhile, serum testosterone significantly (*P* ≤ 0.05) decreased in the AS group when compared with the A group. Moreover, serum testosterone significantly (*P* ≤ 0.05) decreased in the S group when compared with the CM and the control groups. On the other hand, the serum testosterone significantly (*P* ≤ 0.05) decreased in the A and AS groups when compared with the other groups at 28 days; however, the AS group significantly (*P* ≤ 0.05) increased compared with the A group. Regarding results from different periods inside the same group, serum testosterone significantly (*P* ≤ 0.05) decreased in the A group at 28 days when compared with the result at 21 days. Moreover, the results of the other groups significantly (*P* ≤ 0.05) increased at 28 days.

**Table 2:** Effect of camel milk and silymarin on serum testosterone (TESTO) concentration for rats fed on a diet containing 1.4 mg of aflatoxin B1/kg diet for 21 and 28 days (*n* = 5 for each period/group)

Groups items	Day	Control	CM	S	A	ACM	AS
TESTO (ng/dl)	21	2.19 ± 0.08^aY^	2.14 ± 0.08^aY^	1.73 ± 0.21^bY^	1.43 ± 0.06^bX^	1.97 ± 0.06^bX^	1.06 ± 0.16^cX^
	28	2.89 ± 0.21^aX^	2.85 ± 0.05^aX^	2.54 ± 0.13^aX^	1.0 ± 0.04^cY^	2.77 ± 0.29^aY^	1.83 ± 0.10^bY^

Values are means ± SE. Mean values with different subscript letters (a–d) at the same row significantly differ at (*P* ≤ 0.05). Mean values with different subscript letters (X-Y) at the same column significantly differ at (*P* ≤ 0.05). CM: Camel milk; S: silymarin, A: Aflatoxin, ACM: Aflatoxin + camel milk, AS: Aflatoxin + silymarin

**Fig. 1:**
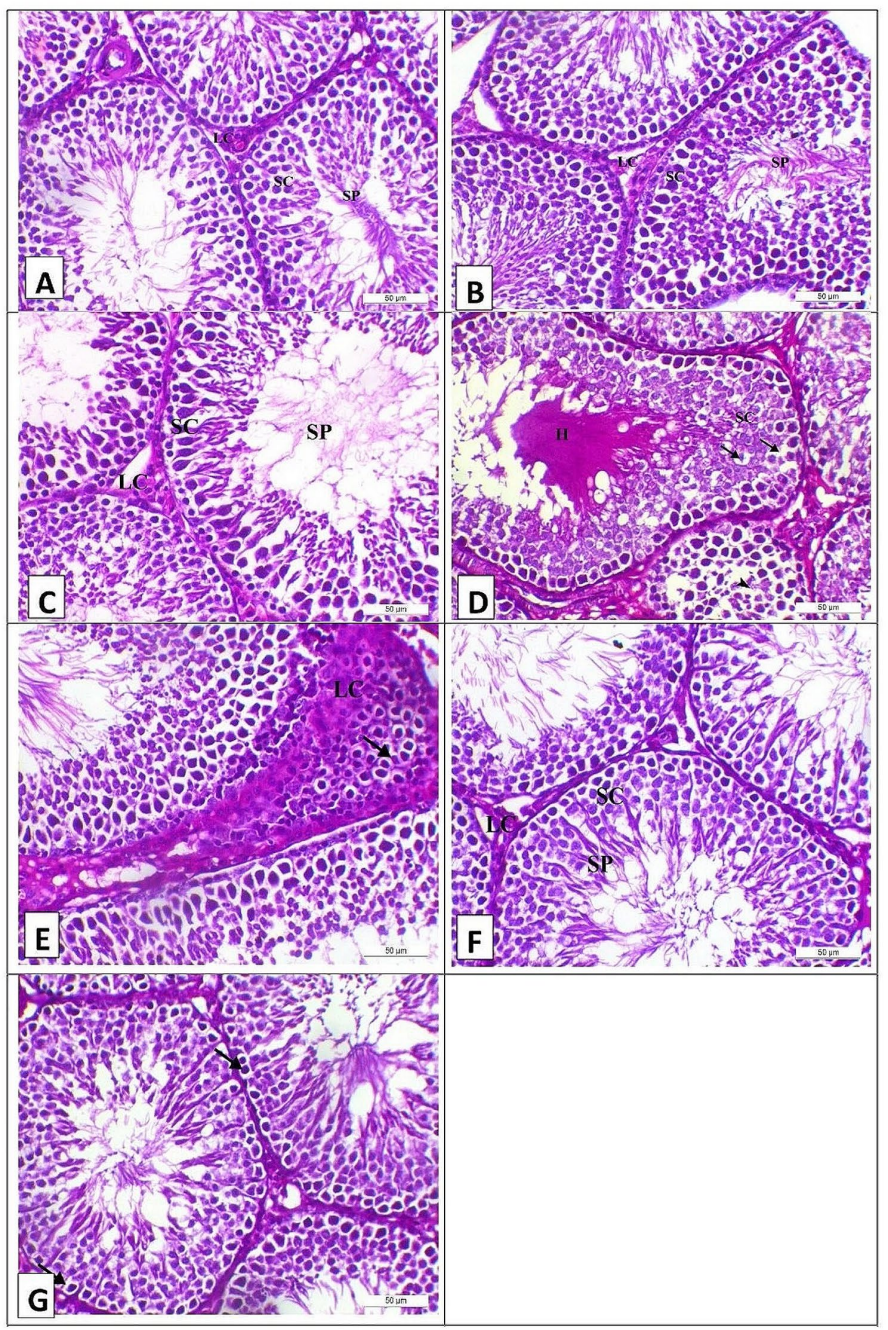
Photomicrograph of the testis of the experimental groups on the 21th day of the experiment, stained with H&E (X200, Scale bar = 50µ) (**A**) Control group showed normal testicular histology as the seminiferous tubule lined with a multilayer of spermatogenic cells (SC) and impacted with sperm (SP) with interstitial Leydig cells (LC). (**B**) Camel milk group showed normal testicular histology as the seminiferous tubule lined with a multilayer of spermatogenic cells (SC) and impacted with sperm (SP) with interstitial Leydig cells (LC). (**C**) Silymarin group showed normal testicular histology as the seminiferous tubule lined with a multilayer of spermatogenic cells (SC) and impacted with sperm (SP) with interstitial Leydig cells (LC). (**D**) Aflatoxin group showed degenerative changes of the lining spermatogenic cells (SC) in the form of vacuoles (arrows) and hyalinization (H). (**E**) Aflatoxin group showed hyperplasia of the interstitial leydig cells with vacuolization of the entire leydig cells (arrow). (**F**) Aflatoxin and camel milk group showed normal testicular histology as the seminiferous tubule lined with a multilayer of spermatogenic cells (SC) and impacted with sperm (SP) with interstitial Leydig cells (LC). (**G**) Aflatoxin and camel mild group showed mild vacuolation of the 1st layer of spermatogenic cells (arrows) with impacted seminiferous tubules with sperm (*n* = 5/group)

**Fig. 2:**
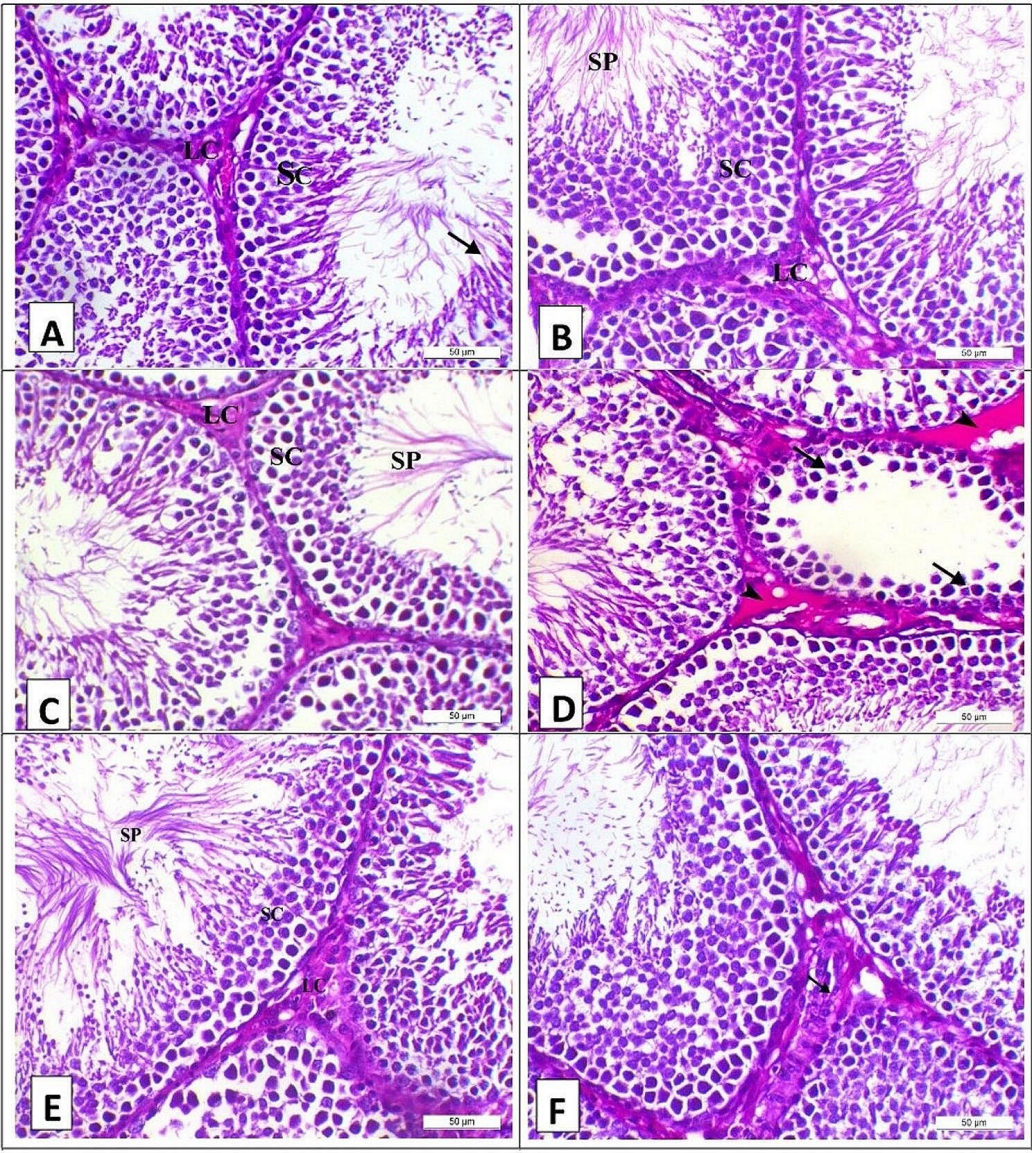
Photomicrograph of the testis of the experimental groups on the 28th day of the experiment, stained with H&E (X200, Scale bar = 50µ) (**A**) Control group showed normal testicular histology as the seminiferous tubule lined with a multilayer of spermatogenic cells (SC) and impacted with sperm (arrow) with interstitial Leydig cells (LC). (**B**) Camel milk group showed normal testicular histology as the seminiferous tubule lined with a multilayer of spermatogenic cells (SC) and impacted with sperm (SP) with interstitial Leydig cells (LC). (**C**) Silymarin group showed normal testicular histology as the seminiferous tubule lined with a multilayer of spermatogenic cells (SC) and impacted with sperm (SP) with interstitial Leydig cells (LC). (**D**) Aflatoxin group showed the arrest of spermatogenesis in some seminiferous tubules (arrows) with degenerative changes of spermatogenic cells and interstitial edema (arrowhead). (**E**) Aflatoxin and camel milk group showed normal testicular histology as the seminiferous tubule lined with a multilayer of spermatogenic cells (SC) and impacted with sperm (SP) with interstitial Leydig cells (LC). (**F**) Aflatoxin and silymarin group showed normal seminiferous tubule with mild hyperplasia of the interstitial Leydig cells (arrow) (*n* = 5/group)

The results of the testicular tissue histopathology are shown in Figs. (1) and (2) at 21 and 28 days, respectively. Photomicrograph of the testis of the control, CM, S, and ACM groups on the 21th day of the experiment showed normal testicular histology as the seminiferous tubule lined with a multilayer of spermatogenic cells (SC) and impacted with sperm (SP) with interstitial Leydig cells (LC) (Fig. 1A, B, C, and F, respectively). Meanwhile, the A group showed degenerative changes of the lining spermatogenic cells (SC) in the form of vacuoles (arrows) and hyalinization (H) (Fig. 1D). Moreover, the A group showed hyperplasia of the interstitial Leydig cells with vacuolization of the entire lying cells (arrow) (Fig. 1E). Meanwhile, the AS group showed mild vacuolation of the 1st layer of spermatogenic cells (arrows) with impacted seminiferous tubules with sperm (Fig. 1G).

Photomicrograph of the testis of the control, CM, S, and ACM groups on the 28th day of the experiment showed normal testicular histology as the seminiferous tubule lined with a multilayer of spermatogenic cells (SC) and impacted with sperm (SP) with interstitial Leydig cells (LC) (Fig. 2A, B, C, and E, respectively). Meanwhile, the A group showed the arrest of spermatogenesis in some seminiferous tubules (arrows) with degenerative changes of spermatogenic cells and interstitial edema (arrowhead) (Fig. 2D). Meanwhile, the AS group showed a normal seminiferous tubule with mild hyperplasia of the interstitial Leydig cells (arrow) (Fig. 2F).

**Fig. 3:**
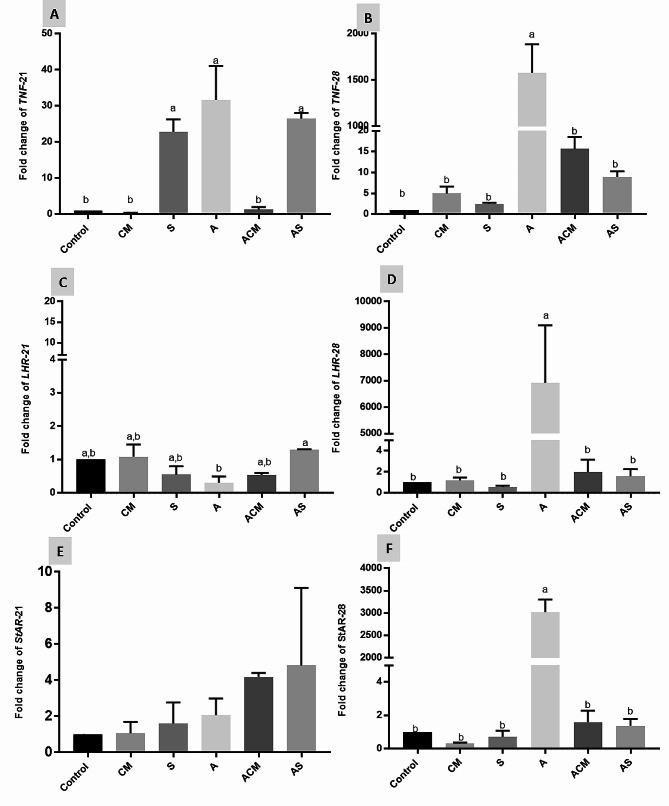
Ameliorative effects of camel milk and silymarin on the gene expression of (**A**) and (**B**) Tumor necrotic factor *α* (*TNFα)*) at 21 and 28 days of treatment respectively, (**C**) and (**D**) Luteinizing hormone receptor (LHR1) at 21 and 28 days of treatment respectively, and (**E**) and (**F**) Steroidogenic acute regulatory protein (StAR) at 21 and 28 days of treatment respectively. CM: Camel milk; S: silymarin, A: Aflatoxin, ACM: Aflatoxin + camel milk, AS: Aflatoxin + silymarin. Values are means ± SE. Mean values with different subscript letters (a-b) significantly differ at (*P* ≤ 0.05). (*n* = 5 for each period/group)

Regarding results of gene expression, qRT-PCR was used to evaluate gene expression levels, with normalization to *β-actin* mRNA at different periods (21 and 28 days), as shown in Fig. 3. At 21 days, aflatoxin administration produced a significant (*P* ≤ 0.05) upregulation of *TNFα* mRNA expression (Fig. 3A), a significant downregulation of *LHR1* (Fig. 3C), and no change in *StAR1* gene expression (Fig. 3E), compared with the control group. Meanwhile, the administration of silymarin with aflatoxin (AS) significantly (*P* ≤ 0.05) upregulated the gene expression of *LHR1*, but there was no significant (*P* ≤ 0.05) upregulation of *StAR1*, and maintained *TNFα* expression level as aflatoxin group. Camel milk with aflatoxin significantly (*P* ≤ 0.05) downregulated *TNFα* in comparison with the levels in the aflatoxin group and non-significantly (*P* ≤ 0.05) upregulated *LHR1* and *StAR1*in comparison with the aflatoxin group. At 28 days, aflatoxin administration produced a significant (*P* ≤ 0.05) upregulation of *TNFα, LHR1*, and *StAR1* mRNA expression (Fig. 3B, D, and F), in comparison with the levels in control untreated rats and other groups. A restored gene expression to the control level was observed in the groups receiving camel milk or silymarin combined with AFB1 (ACM, AS).

## Discussion

From the above-mentioned results, the adverse effects of AFB1 on body weight were mitigated by camel milk and silymarin, which were orally ingested. CM is a great source of biologically active proteins, particularly enzymes with multiple biological functions like antibacterial, antiviral, immune, and antioxidant activity [19]. Mohamed et al. [20] reported that when compared to rats exposed to Fenpropathrin alone, the administration of CM to the CM/Fenpropathrin and Fenpropathrin/CM groups significantly reduced body weight, normalized testis weight, and the gonadosomatic index. The body’s fortification with essential nutrients, including fat, lactose, cholesterol, vitamins, and minerals, may also be responsible for the supportive effects of CM on body weight. Additionally, it possesses defense-enhancing proteins and enzymes with antiviral, antibacterial, and immunological effects [21]. On the other hand, antioxidants in medicinal plants are higher than in fruits and vegetables [22], and studies have shown that these antioxidant characteristics can reduce intestinal adhesions [23]. Their phenolic components improve production efficiency by reducing the number of harmful microorganisms in the stomach and preventing nutrient loss, which improves intestinal health, nutrient digestion, and absorption [24]. Japanese quail receiving a diet containing different levels of milk thistle boosted their feed intake, gained weight, and produced more breast, thighs, and carcass parts [25].

Young animals’ development rate, feed utilization, and immunological response were all slowed down by AFB1. The most significant negative effects on the economy are decreased productivity, impaired immunological response, and pathological changes to organs and tissues [26, 27]. Additionally, aflatoxins (AFs) are thought to be a significant risk factor for male infertility [28, 29].

According to many other researchers [30–32], who reported that body weight in AFB1 mice dramatically dropped when compared to the control group of mice, the body weight statistics from the current study were in great agreement with those studies. AFB1 treatment decreased live body weight and DMI in a dose-dependent manner at 15 and 30 g/kg b.wt [33]. In prior investigations, aflatoxins were given to chickens, cattle, pigs, and rabbits [32, 34–36, respectively], all of which demonstrated a reduction in body weight and DMI. Additionally, rats treated with AFB1 alone showed a substantial decrease in body weight gain and relative organ weight (epididymis, testes, and hypothalamus) compared to the control animals, according to Owumi et al. [37]. On the other hand, according to some studies, AFB1 could change a cell’s energy metabolism by interfering with gluconeogenesis, the tricarboxylic acid cycle, and fatty acid synthesis. These changes could induce the intestinal absorbing barrier and slow the growth of body weight [31]. The fact that AFB1 slowed the growth of the rats may be mostly due to these reports. Depression of appetite may be to blame for the drop in body weight [38]. Aflatoxins were also noted to reduce the formation of volatile fatty acids (VFA), proteolysis, and gastrointestinal tract motility [39].

Leydig cells create testosterone, which is necessary for controlling spermatogenesis and maintaining testicular function [40, 41]. Spermatogenesis is inhibited by the lack of testicular testosterone synthesis in both men and rodents, which lowers sperm density [41]. The results of the current study’s testosterone level analysis were in excellent agreement with those of numerous other researchers. Gad et al. [42] found that camel milk administration increased serum and testicular testosterone in male rats. Moreover, Al-Asha and Arabia [43] found that rats fed CM gained weight in their reproductive organs, had higher sperm counts, and produced more reproductive hormones. These results may be attributed to the large amount of Zn in camel milk [7], where Zn is required to maintain serum testosterone levels [44]. The pituitary gland cannot release luteinizing and follicle stimulating hormones, which promote the synthesis of testosterone, if zinc levels are insufficient [45]. Moreover, Boland [46] reported that, by means of the enzymes that regulate the arachidonic acid cascade, zinc also plays a role in the secretion and functionality of the male hormone testosterone. On the other hand, Abedi et al. [9] found that rat testosterone increased with silymarin and attributed this increase to the antioxidant effect of silymarin. Moreover, Attia et al. [47] found that milk thistle seeds significantly improved semen quality and fertility, and higher testosterone concentration in rabbit bucks. On the other hand, the effect of aflatoxin on testosterone was explained by Salem et al. [33], who attributed the reduction in testosterone secretion to the lower testis weight in the rabbit. Aflatoxins also induced a reduction in the serum testosterone concentrations of White Leghorn cockerels [48]. Furthermore, it was discovered by Mathuria and Verma [49] and Abu El-Saad and Mahmoud [50] that in mice exposed to AFB1, a drop in sperm concentration is followed by a drop in blood testosterone level. Male mice exposed to AFB1 had considerably lower blood testosterone levels [28]. According to Owumi et al. [37], exposure to AFB1 alone caused testicular tissue’s G6PD, LDH, ACP, and ALP activities to decline simultaneously with blood levels of testosterone, FSH, and LH when compared to control rats. The decrease in blood testosterone levels indicates that AFB1 has a suppressive effect on testicular steroidogenesis, which was previously linked to AFB1’s harmful effects on Leydig cells [51, 52]. However, these adverse effects of AFB1 on the testosterone blood level were improved by camel milk and silymarin, which were orally ingested. These results agreed with Mohamed et al. [20], who showed that the levels of sex hormones were elevated more than those in the Fenpropathrin group after CM administration.

The testes, the organs in charge of spermatogenesis, are typically exposed to low oxygen levels due to their poor vascularity. However, because there are so many highly unsaturated fatty acids in the testes, it is known that they are extremely susceptible to oxidative and peroxidative damage [53]. The findings of the testes pathology at different periods are in agreement with the results of Murad et al. [54] and Zhang et al. [55], who find that rats and mice and pigs, respectively, that consumed aflatoxins for an extended period of time experienced histological changes in their male reproductive organs that led to the dystrophy of spermatogenic epithelial cells. Aflatoxin exposure has also been linked to abnormalities in meiotic chromosomes, sperm morphology, and testis histology [56]. Variable levels of seminiferous tubule affection were seen in specimens from animals treated with AFB1. With disorganized germinal epithelium and thickened basement membranes, some tubules shrank. The majority of the spermatogenic and Sertoli cells had pyknotic nuclei and vacuolated cytoplasm [57]. Aboelhassan et al. [58] revealed that, when compared to the control group, the AFB1 therapy caused some seminiferous tubules to degenerate in the testis. This degeneration was identified by a decrease in spermatogenic cells and the buckling of the seminiferous tubule basement membrane. As demonstrated by tubular necrosis of the seminiferous tubules and insufficient sperm in the lumen, the epididymis and testis of AFB1 treated rats displayed considerable modification in their histological architectural framework [59].

However, this effect was mitigated by camel milk and silymarin ingestion. Mohamed et al. [20] reported that prophylactic CM administration improved spermatogenesis activity with the right spermatozoa percentage. Badr et al. [60] reported the effects of camel whey protein in protecting mice’s testes from scrotal heat-induced damage and infertility. Moreover, Faraji et al. [61] reported that the administration of silymarin to the cadmium chloride rats prevented changes in the testicular histological characteristics and testosterone levels in the blood. Rats given silymarin and acrylamide together experienced lessened testicular histological alterations [62]. Silymarin is remarkably similar to steroid hormones, which influence gene expression by boosting protein synthesis [63].

Nitric oxide synthase in the cell is said to be activated by TNF-a control of cytokine production during an inflammatory response. Increased cellular NO levels are linked to nitrosamine stress, which is said to damage cellular proteins, nucleic acids, and lipids as a result of declining antioxidant defense mechanisms [64]. According to Owumi et al. [37], exposure to AFB1 alone significantly decreased levels of the anti-inflammatory cytokine, *IL-10*, in the epididymis, testes, and hypothalamus of the treated rats while significantly increasing levels of *NO, TNF-a*, and *IL-1b*. This finding indicates that inflammation has been induced. Male infertility is well recognized to be brought on by the unchecked production of pro-inflammatory cytokines in the testes, such as *IL-1b* and *TNF-a*, which are damaging to spermatogenesis [65]. At 21 and 28 days in the current study, aflatoxin treatment caused a substantial elevation of *TNF*- gene expression. However, there was a considerable downregulation of *TNF*- gene expression in camel milk and silymarin with aflatoxin.

*StAR* is typically regarded as the rate-limiting step in steroidogenesis and is required for the transport of cholesterols into the mitochondria [66]. The LHR-mediated steroidogenic pathway can control *StAR* expression and activation in Leydig cells [51, 67]. However, Mathuria and Verma [49] and Verma and Mathuria [68] reported that by inhibiting the protein expression of the steroidogenic enzyme, AFB1 prevents the synthesis of testosterone in the testicles, which lowers sperm density. At 21 days, aflatoxin administration produced a downregulation of *StAR1* without being significant in comparison with the control group. These findings support the findings of Cao et al. [28], who found that *StAR, P450scc*, and *17-HSD* protein expression levels were significantly lower in the AFB1 groups than in the control group. AFB1 reduces the expression of several essential steroidogenesis proteins, weakening Leydig cells’ capacity for biosynthesis [52].

Moreover, aflatoxin administration produced a significant downregulation of *LHR1* in comparison with the control group at 21 days. Meanwhile, the administration of camel milk or silymarin with aflatoxin significantly upregulated the gene expression of *LHR1* in comparison with the levels of the control group. These findings corroborated those of Mohammad et al. [69], who noted that LHR expression in the aflatoxin group significantly decreased as compared to the control group, whereas CMA and SA groups compared with the A group. On the other hand, aflatoxin administration showed a significant upregulation of *LHR1* in comparison with the control group at 28 days. These results agree with the findings, which revealed up-regulation of gene expression of the *LHR* gene in rats that were exposed to AFB1 as compared to those found in the normal control [58].

## Conclusion

Overall, it was observed that there were many different beneficial effects resulting from the camel milk and silymarin used in this trial. Camel milk and silymarin induced positive effects on growth, reproductive organs, and gene expression of the tumor necrosis factor α, luteinizing hormone receptor, and steroidogenic acute regulatory protein with normal testicular architecture. On the contrary, the presence of aflatoxin in the feed of rats affected the above-mentioned parameters. However, camel milk and silymarin may be able to counteract the detrimental effects of AFB1 on rat body weight and reproductive organs.

## Materials and methods

### Experimental animals and design

This study was approved by the Ethics of Animal Use in Research Committee (IACUC), Faculty of Veterinary Medicine, Alexandria University, Egypt (No. 0304593). To conduct this research, a total of sixty mature male Wister white rats (4 weeks old), each weighing an average of 83.67 ± 0.21 gm, were used. The animals were obtained from a private farm (Abdo Farm for Lab. Animals), Alexandria, Egypt. The authors had received consent to use the rats after officially applying to the Faculty of Veterinary Medicine, Matrouh University. All rats were housed in 70 × 50 × 30-centimeter ploy propylene cages with the temperature set to 19–22°C to maintain a pathogen-free environment. With a 12-hour cycle of light and nighttime, the relative humidity was close to 60%. The water was supplied *ad libitum* during the experimental period and two weeks of acclimatization. There were six groups of rats, each with ten animals. The groups were the control (received saline orally), CM (1 ml of camel milk/kg body weight orally, Gad et al. [42]), S (20 mg silymarin/kg b. wt. suspension, orally, Rastogi et al. [70]), A (1.4 mg aflatoxin/kg diet, El-Nekeety et al. [71]), ACM (aflatoxin plus camel milk), and AS (aflatoxin plus silymarin). The control and A groups received saline orally. All treatments were given for 21 and 28-days at 10 AM. All animal handling procedures are in agreement with the ARRIVE guidelines from the National Center for the Replacement, Refinement, and Reduction of Animals in Research (NC3Rs) ^19^ throughout the experimental period (28 days). Also, all methods were carried out in accordance with relevant guidelines and regulations.

### Drugs and chemicals

From the Mycology Division of the Animal Health Research Institute in Giza, Egypt, aflatoxin was acquired. From Egypt’s Marsa Matrouh, we bought camel milk at a neighborhood bazaar. Bottles were used to carry the camel milk to the lab, where it was kept chilled. Silymarin was acquired from Hepamarin capsules (140 mg silymarin/capsule), (uni pharma).

### Body weights

To determine the body weight of rats, they were weighted individually after acclimatization period (at the start of the experiment) and at 21 days and 28 days from the start (end of the experiment). A gram scale was calibrated before the weight measurements according to facility standard operating procedures.

### Sampling

Following treatments of 21 and 28 days, samples from all groups were collected after overnight fasting. 1.5% Isoflurane inhalation [72] was used to anaesthetize the rats before blood samples were taken from the medial canthus of the eye and placed in a sterile vacutainer container without an anticoagulant to separate the serum. The blood samples were kept at a temperature of −20°C until they were used for chemical analysis. Rats were killed by cervical dislocation after sample collection. The testicular tissue was cleaned in saline and split into two pieces; one was preserved in formalin for histopathological analysis while the other was kept at −80°C pending RNA isolation.

### Biochemical analysis

Serum total testosterone concentration was determined by a Microplate Enzyme Immunoassay, colorimetric according to Dorfman and Shipley [73] by using ab285350 – Mouse/Rat Testosterone ELISA Kit-Abacam, USA.

### Histological processing of the testes

The testicular tissue (5 testes for each period/group) was fixed in 10% neutral buffered formalin. The fixed organ was dehydrated in ascending concentrations of alcohol from 70% until reaching absolute alcohol, cleared using multiple changes of xylene, wax impregnated using paraffin wax, and embedded in paraffin wax using a mold of suitable size. Paraffin blocks containing specimens were then cut into thin Sect. (5 µM each) by using a microtome, and selected sections were mounted on glass slides. The preparation of paraffin sections for staining included the removal of paraffin wax by using xylene, the removal of xylene by the use of absolute alcohol, treatment with descending concentration of alcohol (from absolute untill 70%), and washing the sections with distilled water. Sections were stained by “Hematoxylin and Eosin” (H&E) stain and examined under a light microscope [74].

### Gene expression

The Quantitative real-time PCR (qRT-PCR) was used to determine the expression of *TNFα*, tumor necrosis factor α; *LHR*, luteinizing hormone receptor; *StAR*, steroidogenic acute regulatory protein. The testicular samples (*n* = 5) were collected, frozen in liquid nitrogen, and stored at −80°C for further RNA extraction. Following the manufacturer’s instructions, RNA was extracted from the frozen samples using an RNA Purification Kit from Thermo Scientific (USA). The Intron-Power cDNA synthesis kit (Thermo Scientific, USA) was used to create cDNA from a fixed concentration of RNA. For the qRT-PCR assay: specific primers were used to amplify *TNFα*, *LHR*, and *StAR*, in rats, with the β-actin housekeeping gene for normalization. The primers are presented in Table 3. The obtained data were analyzed using the 2^−ΔΔCt^ method [75].

**Table 3:** Primers used in this study

Genes	Primer’s sequence (5’–3’)	Acc. number
*TNFα*	F: CCACGTCGTAGCAAACCACCAAG R: CAGGTACATGGGCTCATACC	NM_012675.3 [76]
*LHR*	F: CCAGAACACCAAAAACCTGCT R: ATCTGGAAGGGTTCGGATGC	NM_012978 [77]
*StAR*	F: GCCTGCAATTTGGTGGA R: GGGCATACTCAACAACCAG	NM_031558 [78]
*β-actin*	F: CACCATGTACCCAGGCATTG R: ACAGTCCGCCTAGAAGCATT	NM_031144.3 [79]

*TNFα*, tumor necrosis factor α; *LHR*, luteinizing hormone receptor; *StAR*, steroidogenic acute regulatory protein

### Statistical analysis

The findings were shown as mean ± standard error of the mean (SEM). To compare statistical data, a one-way analysis of variance (ANOVA) was performed. Utilizing GraphPad Prism version 7.00 for Windows, GraphPad Software, La Jolla, California, USA, www.graphpad.com, statistical analysis and graphs were created. Significant was located at *P* ≤ 0.05.

## Data Availability

The datasets used and/or analyzed during the current study are available from the corresponding author upon reasonable request.

## Declarations

### Guidelines

All methods were carried out in accordance with relevant guidelines and regulations.

### ARRIVE guidelines

The authors confirm that the study was carried out in compliance with the ARRIVE guidelines.
